# When complexity matters: a step-by-step guide to incorporating a complexity perspective in guideline development for public health and health system interventions

**DOI:** 10.1186/s12874-020-01132-6

**Published:** 2020-10-02

**Authors:** A. Movsisyan, E. Rehfuess, S. L. Norris

**Affiliations:** 1 Institute for Medical Information Processing, Biometry and Epidemiology, LMU Munich, Marchioninistrasse 17, 81377 Munich, Germany; 2 Pettenkofer School of Public Health, LMU Munich, Marchioninistrasse 17, 81377 Munich, Germany; 3 Science Division, Department of Quality Assurance of Norms and Standards, World Health Organization, 20 Avenue Appia, 1211, 27 Geneva, Switzerland

**Keywords:** Guideline, Recommendation, Systematic reviews, Complexity perspective, Systems thinking, Logic model, Decision criteria, Stakeholder

## Abstract

**Background:**

Guidelines on public health and health system interventions often involve considerations beyond effectiveness and safety to account for the impact that these interventions have on the wider systems in which they are implemented. This paper describes how a complexity perspective may be adopted in guideline development to facilitate a more nuanced consideration of a range of factors pertinent to decisions regarding public health and health system interventions. These factors include acceptability and feasibility, and societal, economic, and equity and equality implications of interventions.

**Main message:**

A 5-step process describes how to incorporate a complexity perspective in guideline development with examples to illustrate each step. The steps include: (i) guideline scoping, (ii) formulating questions, (iii) retrieving and synthesising evidence, (iv) assessing the evidence, and (v) developing recommendations. Guideline scoping using stakeholder consultations, complexity features, evidence mapping, logic modelling, and explicit decision criteria is emphasised as a key step that informs all subsequent steps.

**Conclusions:**

Through explicit consideration of a range of factors and enhanced understanding of the specific circumstances in which interventions work, a complexity perspective can yield guidelines with better informed recommendations and facilitate local adaptation and implementation. Further work will need to look into the methods of collecting and assessing different types of evidence beyond effectiveness and develop procedural guidance for prioritising across a range of decision criteria.

## Background

Guidelines are a key instrument in clinical practice, public health, and health system decision-making and offer recommendations on how to choose among different interventions and policies to improve health. Development of clinical practice guidelines follows a systematic and transparent process primarily focusing on and prioritising questions about the health effects of interventions [1]. However, decisions on public health and health system interventions often need to consider broader questions beyond effectiveness and safety [2]. These interventions tackle a range of behavioural, social, commercial, political, and environmental determinants of health and are implemented in complex systems with specific contextual features [3, 4]. Here, guidelines usually need to consider more nuanced questions on why, how, and in what circumstances these interventions work and what their impact on the wider system might be.

The phrase *complex intervention* has often been used to describe public health and health system interventions, which: (i) have many interacting components; (ii) involve complex behaviours during their delivery and receipt; (iii) target different groups and levels; (iv) influence many health and non-health outcomes; or (v) require flexible implementation across different contexts [5, 16]. *Complex systems*, on the other hand, refer to the dynamic networks of social interactions in which interventions take place [17, 18]. In fact, interventions interact with and influence the wider systems in which they are delivered regardless of whether the interventions themselves may be simple or complex in design (e.g., a drug or a multi-component chronic disease management programme) [9]. Smoke-free legislation provides an example of a simple intervention in design. However, the introduction of smoke-free legislation initiates complex system changes through its impact not only on the smoking-related health outcomes, but also on the patterns of socialising and drinking in the community [5, 19]. In this paper, we use the term *complexity perspective* to refer to the broad changes that interventions bring into the dynamic systems in which they are delivered regardless of their design features. To operationalise this perspective, we highlight key aspects of complexity derived from complex systems theory and illustrate them in the context of public health and health system interventions (see Table 1).

**Table 1:** Aspects of complexity to consider in developing guidelines on public health and health system interventions (adapted from Petticrew and colleagues [5] and Rehfuess and colleagues [6])

Aspects of complexity	Description	Example
Interactions between intervention components	Interventions may include multiple interacting components which may have synergistic or dysynergistic effects on the system.	Parenting interventions to prevent child maltreatment may include multiple components relating to parenting knowledge, skills and parental mental health to bring about changes in the family environment and parent-child relationships [7].
Interaction of interventions with context	Interventions – and their components – may be context-dependent, i.e. their effectiveness, feasibility and acceptability may be affected by the epidemiological, socio-economic, socio-cultural, political, legal and other characteristics of a given context.	Giving corticosteroids to women at risk of pre-term delivery can effectively reduce the risk of fetal and neonatal deaths in hospitals with special care in high-income countries; in countries without such special care hospitals antenatal corticosteroid therapy may do more harm than good [8].
Dynamism	Systems evolve and change over time as a result of interactions among diverse agents.	School teachers, staff and students constantly change within schools [9].
Adaptivity and co-evolution	Interventions may influence the context of implementation (directly or indirectly). The entire system adapts and responds in expected or unexpected ways. The interventions themselves also change in response to system changes.	Regulations to ban smoking in public places or to prohibit the sale of tobacco products with certain characteristics may affect individual consumption; manufacturers may reformulate tobacco products as a response. This may further change how regulations are formulated or implemented [10].
Emergent properties	Intervention effects may emerge from self-organisation among the interacting agents.	Herd immunity is an emerging effect of human papillomavirus vaccination of a sufficient percentage of the population [11].
Non-linearity and phase changes	Interventions may demonstrate effects once they have reached a certain scale.	Community sanitation interventions first need to reach thresholds in the order of 60% or higher, to optimise health and nutrition gains [12].
Feedback loops	Interventions comprised of different components can produce feedback loops reducing the overall effect (negative), or conversely, enhancing the effect beyond what might be expected (positive).	Interventions to increase the availability of healthy foods promote healthy diets, which further enhance the need for healthy foods (positive feedback loop) [13].
Multiple (health and non-health) outcomes and dependencies	Interventions such as those involving multiple components often impact a large number of health and non-health outcomes and involve complex causal pathways.	In addition to the direct effects of alcohol advertising restrictions on consumption, such restrictions may also affect non-health outcomes, such as spending on alcohol, risk behaviours, and social norms around consumption [14, 15].

We believe that a complexity perspective facilitates a more nuanced consideration of a range of questions that are pertinent to decisions regarding public health and health system interventions (see Table 1). These include questions on the impact of the intervention on the broader system, including, for example, how an intervention interacts with a specific socio-economic and cultural context and what health and non-health outcomes it affects. Ultimately, this enables guideline recommendations that are driven not primarily by considerations of effectiveness and safety in relation to a narrow set of health outcomes, but equally by factors such as acceptability, feasibility, societal implications, and equity and equality [6]. Taking a complexity perspective in guideline development therefore helps make better informed recommendations based on a comprehensive understanding of the intervention and its manifold implications for the system. Importantly, such a perspective helps avoid simplistic and misleading guideline recommendations, which may ignore critical contextual features affecting the benefits and harms, acceptability or feasibility of an intervention or running counter to relevant social or environmental considerations [5]. The introduction of a levy on soft drinks to reduce obesity can serve as an illustrative case. A standard approach would be based on a linear model of cause and effect, where the introduction of the levy is expected to reduce the purchase and consumption of soft drinks, ultimately resulting in lower rates of obesity among children and the general population. On the other hand, a complexity perspective would encourage asking questions regarding the entire system, including access to and safety of healthier alternatives, notably water, and likely reactions from the concerned industries, such as changes to the composition, pricing, and marketing of various commodities [20]. The recommendations of a guideline taking a complexity perspective on obesity may therefore be different from those reached by assuming and examining a linear cause-effect relationship [14].

The approach described in this paper builds upon and extends a series published in *BMJ Global Health* on *Complex health interventions in complex systems: improving the process and methods for evidence-informed health decisions*, as well as other selected methodological work on developing guidelines and considering complexity in evidence synthesis published in the last decade [2, 21–24]. The series was specifically commissioned by the World Health Organization (WHO) to strengthen its methods for guideline development. It involved convening working groups and consensus meetings with leading international experts in systematic review methodology, guideline development and complex systems thinking. The papers in the series focus on key concepts of complexity and their implications for guideline development and were developed using a range of methods, including systematic and non-systematic literature reviews [2].

**Fig. 1:**
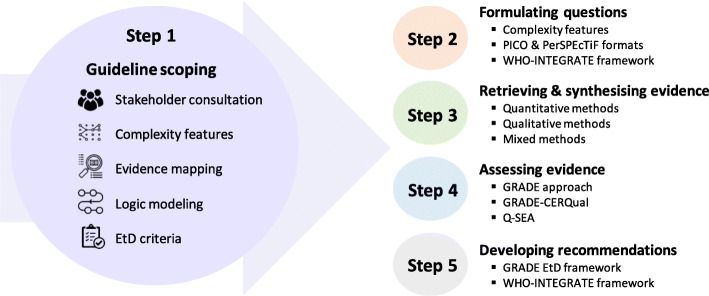
Incorporating a complexity perspective into the process of guideline development. The diagram shows how a complexity perspective may be incorporated in each step of guideline development. It emphasises guideline scoping as a key step that informs all subsequent steps Notes: CERQual, Confidence in the Evidence from Reviews of Qualitative Research; EtD, Evidence to Decision; GRADE, Grading of Recommendations Assessment, Development, and Evaluation; PerSPEcTiF, Perspective, Setting, Phenomenon of interest, Environment, Comparison, Time, and Findings; PICO, Population/Problem, Intervention, Comparison, Outcome; Q-SEA, Quality Standards for Ethics Analyses; WHO-INTEGRATE, World Health Organization INTEGRATe Evidence

In this paper we suggest a five-step process for guideline development when taking a complexity perspective (see Fig. 1): (i) guideline scoping, (ii) formulating questions, (iii) retrieving and synthesising evidence, (iv) assessing the evidence, and (v) developing recommendations. For each step, we explain specific methods to incorporate a complexity perspective and illustrate these by referring back to the above case of introducing a levy on soft drinks to reduce obesity. We then provide an example of how these methods have been applied in existing guidelines. Our aim is to help those involved in guideline development better understand and address the key aspects and challenges of taking a complexity perspective.

## Main text

### Step 1: guideline scoping

Whether to take a complexity perspective is a decision that should be made by considering the topic and aims of a guideline and the needs of guideline users. Taking a complexity perspective entails examination of a broader range of questions in guidelines, which may require additional time and resources. In this early phase, this can be facilitated by stakeholder consultations, examination of the relevant aspects of complexity, logic modelling, evidence mapping, and a review of Evidence to Decision (EtD) criteria. Guideline panels should choose the most suitable procedure or several among these. As illustrated in Fig. 1, using these procedures during scoping will inform all other steps in the guideline development process. In addition, some of these procedures can also be employed in subsequent steps.

While stakeholder consultation is commonly used in guideline development and increasingly in systematic reviews [25–27], it is particularly important when taking a complexity perspective and is relevant to all steps in the development process. Stakeholders can add valuable insights on the optimal scope, and input into setting priorities among a range of key questions regarding the interactions of the intervention with the system. For public health and health system interventions, key stakeholders include guideline end-users, such as providers and organisations delivering or financing the intervention and those directly affected by the guideline recommendations, such as specific population groups, industry, or the general public.

The means of involving stakeholders and their level of engagement in guideline development will depend on the topic, the stakeholders concerned, and feasibility considerations [5]. For example, if a guideline includes children or adolescents as key stakeholders, it would be more appropriate to elicit their views using qualitative or participatory research than to invite them to join the guideline panels and sit through panel meetings [28]. During guideline scoping, stakeholders can help define guideline priorities, relevant questions and contextual factors, and the key areas of uncertainty that need to be explored, for example, with new systematic reviews. The views of relevant stakeholders can be incorporated via their direct involvement in the guideline panel, through surveys or needs assessments or through primary or secondary research on their views. The TRANSFER Approach can be used to facilitate stakeholder input into contextual features from the beginning of the systematic review and – by extension – the guideline development process [27]. It enables a stakeholder-driven systematic and transparent assessment of transferability factors using a structured process.

The aspects of complexity outlined in Table 1 provide guideline panels with key concepts that can help with identifying whether taking a complexity perspective is warranted, as well as with scoping the guideline and related decisions regarding relevant aspects of complexity. Consideration of these concepts in relation to the guideline topic and interventions can highlight key questions to prioritise in the guideline [5]. This may be achieved by formulating and answering questions, such as (i) does the intervention of interest include many interacting components (e.g., different technologies and behaviour change activities as part of a sanitation intervention)? (ii) Does the intervention interact with and change the context into which it is introduced (e.g., changing social norms in addition to health outcomes in case of smoke-free legislation)? (iii) Does the intervention operate through system-level mechanisms (e.g., in order to change substance use outcomes in students, the entire school ethos might need to be transformed). Positive answers to these questions suggest that there is an added value in prioritising guideline questions that address aspects of complexity.

Logic models provide another useful approach to facilitate decisions on taking a complexity perspective [5, 29, 30]. They graphically display the intervention, different elements of the system and the relationships among them, including known or presumed pathways from the intervention to its various health and non-health outcomes. Two broad types of logic models are distinguished: system-based and process-oriented [30]. System-based logic models, also referred to as conceptual frameworks and sometimes executed as causal loop diagrams, attempt to display the broad system in which the intervention is embedded, including contextual and implementation elements. Developing a system-based logic model can help guideline panels understand and prioritize aspects of complexity such as whether the guideline should only focus on intervention effects in relation to specific health outcomes or whether it should consider questions around intervention implementation. For example, when considering the effects of a levy on soft drinks to reduce obesity, developing a logic model which maps different elements of the system can help explicate how the industry might react to the levy by reformulating sugar in existing products (the intended impact) or by innovating and diversifying their product ranges (an unintended impact). This may inform the guideline panel that system adaptivity might be an important aspect of complexity to explore (see Table 1).

Process-oriented logic models, on the other hand, display the linear or non-linear pathways that lead from the intervention to multiple outcomes considering the temporal sequence of events [30]. Such logic models can facilitate identification of key health and non-health outcomes, feedback loops and phase changes (see Table 1), however they are difficult to design and detailed evidence on pathways is often lacking. In general, logic models can be developed through a combination of searches of the literature and consultations with stakeholders, such as members of the guideline panel and content experts.

Evidence maps are also increasingly used in guideline scoping to obtain an overview of the existing evidence and thereby inform decisions on which aspects of complexity to consider in the guideline. Evidence maps involve systematic searches of a broad field to provide an overview and identify gaps in the evidence and/or future research needs [31]. They often present the results in a user-friendly format, including visual graphs or searchable databases. Evidence maps can draw on a variety of synthesis products or individual studies, for example, existing systematic reviews of effectiveness, qualitative evidence syntheses of factors concerning intervention acceptability, or modelling studies of the various costs and societal benefits of the intervention [31]. Through broad searches of the topic area, evidence maps can help panels decide on guideline priorities and questions and choose efficient methodological approaches to address them. Use of evidence maps can be particularly helpful in scoping when guidelines can draw on a well-developed evidence base – by summarising that evidence base and highlighting areas for further research.

EtD frameworks can also be used to help define guideline priorities, including important aspects of complexity, determine where systematic reviews are indicated, and add transparency in guideline development. They specify a set of criteria that inform the formulation of guideline recommendations, their direction and strength. For example, the EtD frameworks developed by the Grading of Recommendations Assessment, Development, and Evaluation (GRADE) Working Group highlight the priority of the problem, balance of benefits and harms, values and preferences, quality of evidence, resource implications, equity and human rights, acceptability and feasibility [52]. However, currently these factors are usually considered at the end of the guideline development process as supplementary pieces of information to shape recommendations, which are largely driven by evidence on intervention effectiveness. WHO-INTEGRATE (Word Health Organization INTEGRATe Evidence) is another EtD framework that is well-suited for guidelines taking a complexity perspective. Adopting a societal perspective, it describes a broad set of criteria and specific sub-criteria to consider during guideline scoping, suggests an explicit and flexible approach towards weighting the importance of these criteria, and promotes a plurality of methods with different types of evidence collected and assessed for each criterion (see Table 2) [6]. Reflecting upon and choosing among these criteria and sub-criteria for in-depth consideration at an early phase of guideline development will help explicate guideline priorities and identify types of evidence that should be consulted to develop recommendations (see Step 5).

**Table 2:** WHO-INTEGRATE framework and suggested methods for evidence synthesis and assessment of quality of evidence (adapted from Rehfuess and colleagues) [6]

Criterion and definition	Sub-criteria	Evidence synthesis methods	Approaches to assessing quality of evidence
**Balance of health benefits and harms** The balance of health benefits and harms reflects the magnitude and types of health impact of an intervention on individuals or populations, taking into account how those affected value different health outcomes.	• Efficacy or effectiveness on health of individuals • Effectiveness or impact on health of a population • Patients’/beneficiaries’ values in relation to health outcomes • Safety-risk-profile of the intervention • Broader positive or negative health-related impacts	• Systematic reviews of efficacy/effectiveness for anticipated effects [32] • Qualitative evidence syntheses [33, 34] and mixed-method reviews [35] or cross-sectional studies [36] for patients’/beneficiaries’ values in relation to health outcomes • Scoping reviews for unanticipated effects [37]	• GRADE [38] • GRADE CERQual (where applicable) [39]
**Human rights and sociocultural acceptability** This criterion encompasses two distinct constructs: The first refers to an intervention’s compliance with universal human rights standards and other considerations laid out in international human rights law beyond the right to health (as the right to health provides the basis of other criteria and sub-criteria in this framework). The second, sociocultural acceptability, is highly time- and context-specific and reflects the extent to which those implementing or benefiting from an intervention as well as other relevant stakeholder groups consider it to be appropriate, based on anticipated or experienced cognitive and emotional responses to the intervention.	• Accordance with universal human rights standards • Socio-cultural acceptability of intervention by patients/ beneficiaries and those implementing the intervention • Socio-cultural acceptability of intervention by the public and other relevant stakeholder groups • Impact on autonomy of concerned stakeholders • Intrusiveness of intervention	• Ethics syntheses [40, 41] for accordance with universal human rights standards • Qualitative evidence syntheses [33, 34, 42] and mixed-method reviews [35] for socio-cultural acceptability and impact on autonomy of concerned stakeholders and intrusiveness of interventions	• GRADE CERQual (where applicable) [39] • Q-SEA for ethics analyses [43]
**Health equity, equality and non-discrimination** Health equity and equality reflect a concerted and sustained effort to improve health for individuals across all populations, and to reduce avoidable systematic differences in how health and its determinants are distributed. Equality is linked to the legal principle of non-discrimination, which is designed to ensure that individuals or population groups do not experience discrimination on the basis of their sex, age, ethnicity, culture or language, sexual orientation or gender identity, disability status, education, socioeconomic status, place of residence or any other characteristics.	• Impact on health equality and/or health equity • Distribution of benefits and harms of the intervention • Affordability of the intervention • Accessibility of the intervention • Severity and/or rarity of the condition • Lack of a suitable alternative	• Quantitative systematic reviews [44] using PROGRESS or PROGRESS PLUS [45, 46], where possible using pre-specified sub-group analyses • Quantitative systematic reviews targeting disadvantaged groups • Equity weights and social welfare functions in economic analyses (*see Financial and economic considerations*). • Qualitative evidence syntheses [33, 34, 42] and mixed-method reviews [35] • Ethics syntheses [40, 41]	• No standardised approach • GRADE for subgroup analyses (where applicable) [38] • Relevant considerations, such as including health equity as an outcome, in Welch et al. [47]
**Societal implications** Societal implications recognise that health interventions do not take place in isolation and may enhance or inhibit broader social, environmental or economic goals in the short or long term. It also reflects the fact that many regulatory, environmental or other population-level health interventions are directly aimed at system-level rather than individual-level changes.	• Social impact • Environmental impact	• Systematic reviews of effectiveness [44] • Qualitative evidence syntheses [33, 42] • Mixed-method reviews [35] • Health technology assessments [48]	• No standardised approach • GRADE (where applicable) [38]
**Financial and economic considerations** Financial and economic considerations acknowledge that available financial (budgetary) resources are constrained and take into account the economic impact of an intervention on the health system, government or society as a whole.	• Financial impact • Impact on economy • Comparison of costs to benefits	• Comprehensive or representative cost or budget impact data at the appropriate level (global, regional, national, sub-national) • Economic burden of disease studies undertaken at the appropriate level (global, regional, national, sub-national). • Economic analyses undertaken at the appropriate level [49] or economic analysis reviews [50]	• No standardised approach • Relevant considerations in Drummond et al. [49] and Brunetti et al. [51]
**Feasibility and health system** Feasibility and health system considerations recognise that the most appropriate and feasible interventions may vary significantly across different contexts, both across countries and across jurisdictions within countries. Legislation and governance, the structure of the health system and existing programmes as well as human resources and infrastructure should be taken into account.	• Legislation • Leadership and governance • Interaction with and impact on health system • Need for, usage of and impact on health workforce and human resources • Need for, usage of and impact on infrastructure	• Qualitative evidence syntheses [33, 42] • Mixed-method reviews [35]	• No standardised approach • GRADE CERQual (where applicable) [39]
**Quality of evidence** Quality of evidence reflects the confidence that the available evidence is adequate to support a recommendation. Quality of evidence is a meta-criterion that can be applied across all criteria in the WHO-INTEGRATE framework (see approaches to assessing quality of evidence).	–	–	–

Notes: *CERQual* Confidence in the Evidence from Reviews of Qualitative research, *GRADE* Grading of Recommendations Assessment, Development, and Evaluation, *PROGRESS PLUS* Place of Residence, Race, Occupation, Gender/sex, Religion, Socioeconomic Status, *Q-SEA* Quality Standards for Ethics Analyses, *WHO-INTEGRATE* World Health Organization INTEGRATe Evidence

#### Illustration from a guideline

To better address the sexual and reproductive health and human rights (SRHR) of women living with HIV, a World Health Organization (WHO) guideline was developed [53]. To scope and structure the guideline from a complexity perspective, a preliminary literature review was conducted, which mapped the evidence on the consideration of human rights in sexual and reproductive health programmes. Based on this evidence map and a global survey to assess SRHR priorities of women living with HIV, a decision was made to structure the guideline explicitly following a woman-centred approach and to uphold the principles of human rights and gender equality. The findings from the survey were also used to draft the guideline questions and inform the recommendations and accompanying remarks in the guideline [53].

### Step 2: formulating questions

Taking a complexity perspective will affect both the types of questions asked and how these questions are formulated. Typically, guidelines prioritise and/or are limited to questions of effectiveness. Often these are formulated as broad questions asking whether an intervention works compared with an alternative intervention, following the PICO (Population, Intervention, Comparison, Outcome) framework [1]. However, public health and health system interventions affect the system in which they are implemented in multiple ways; how they operate and which effects they can achieve depend on a combination of geographical, epidemiological, socio-cultural, socio-economic, political, ethical, and legal factors [54, 55]. It is therefore important for guidelines taking a complexity perspective to more carefully attend to these aspects of complexity in formulating questions. For example, broadly defined PICO elements can be further dissected into sub-questions for description and quantitative examination, where possible [56] (see Step 3): What are the effects of the intervention across different population groups (dissecting the “P” element)? What is the independent effect of a given individual component of the intervention (dissecting the “I” element)? What are the effects of the intervention as assessed by different outcome measures (dissecting the “O” element)? Variation of intervention effects across different contextual characteristics would also be important to explore and assess through quantitative analyses, where data allow.

Questions that extend beyond intervention effectiveness can be formulated using frameworks other than PICO. For example, PerSPEcTiF (Perspective, Setting, Phenomenon of interest, Environment, Comparison, Time, and Findings) can be used to formulate guideline questions related to stakeholder experiences with the intervention in a specific context (see Table 3) [54]. The criteria and sub-criteria of the WHO-INTEGRATE framework can suggest specific guideline questions (see Additional file 1) [6]. This could include, for example: to what extent do stakeholders value different health outcomes (benefits and harms)? What are their views about the acceptability of and preferences regarding the intervention (socio-cultural acceptability)? How will the intervention impact health expenditures, equity, equality, and non-discrimination? What is the ecological impact of the intervention (societal implications)? What is the cost of the intervention (financial impact)? What aspects of the health system influence implementation of the intervention (feasibility and health system considerations)?

**Table 3:** Worked example of a guideline question formulation using the PerSPEcTiF framework (adapted from Booth and colleagues) [42]

Per	S	P	E	(C)	Ti	F
Perspective	Setting	Phenomenon of interest or problem	Environment	Comparison (optional)	Time / timing	Findings
From the perspective of a pregnant woman	In the setting of rural communities	How does the phenomenon of facility-based care	Within an environment of poor transport, infrastructure and geographically remote facilities	Compare with traditional birth attendants at home	In the time period up to and including childbirth	In relation to the woman’s perceptions and experiences?

Stakeholder consultations, logic modelling, and evidence mapping can also help inform question formulation by identifying relevant aspects of the intervention, context, and outcomes (see Step 1) [6, 57]. As presented above, development of a system-based logic model on the potential effects of a levy on soft drinks could help identify important aspect of complexity, such as system adaptivity (see Table 1). This would then inform the following key question: *how might the system change when a levy is imposed on the soft drink industry?*

#### Illustration from a guideline

To address aspects of complexity, the guideline panel developing recommendations on antenatal care (ANC) for a positive pregnancy experience first conducted a scoping exercise to identify and map existing guidelines related to ANC [58, 59]. This highlighted the need to identify women-centred interventions and outcomes for ANC. To this end, a qualitative systematic review was conducted to explore women’s needs and values in pregnancy and ANC. This revealed *positive pregnancy experience* as the primary outcome. The scoping process and stakeholder consultation also led to identification of guideline priority questions and outcomes related to the effectiveness of interventions for a positive pregnancy experience. Examples of the specific questions include the following: for pregnant women (P), do diet and/or exercise interventions (I) compared with standard ANC (C) improve maternal and perinatal outcomes (O) (an effectiveness question regarding a nutritional intervention)? Should pregnant women carry their own ANC case notes to improve quality of care (a question regarding a delivery of a health system intervention)?

### Step 3: retrieving and synthesising evidence

Standard approaches to evidence retrieval and synthesis can be used to explore the details of the guideline PICO questions and contextual variations of the effect [56]. For example, subgroup analyses and meta-regression can be used to explore variation of intervention effects across different contexts and population groups [32]. A component-level approach and network meta-analysis can be used to examine the effects of individual components of the intervention or their combinations [60]. Having few primary studies and many sources of variation can, however, jeopardise the validity of these methods. It is therefore important that plausible sources of diversity are pre-specified in a guideline [56], and logic modelling and stakeholder consultations during guideline scoping may be helpful (see Step 1). A more iterative and flexible process may also be needed to identify and explore these sources. In this case, changes are made to the guideline development protocol as the panel identifies relevant aspects of complexity. These changes should be explicitly documented and the rationale provided. In the case of few primary studies, qualitative comparative analysis (QCA) can be used involving cross-tabulation of evidence to identify configurations of interventions and various contextual factors that may explain the effects [61]. When studies are too diverse to combine in a meta-analysis, findings can be synthesised and reported in a narrative manner [62], and graphical displays (e.g., harvest, forest, albatross, or bubble plots) can be used to illustrate patterns in the data [56]. When effect size estimates are not reported, other information from each study can be used for statistical inferences, such as the direction of effect [56].

Evidence on questions beyond intervention effectiveness can be synthesised using quantitative, qualitative, and mixed methods synthesis. Quantitative approaches include model-driven meta-analysis, which can be used to explore intervention mechanisms driving the overall effect [56]. Model-based approaches can also be used to examine how the wider system changes with intervention implementation. These approaches can be viewed as mathematical representations of (often simplified) logic models and may include empirical data (e.g., from systematic reviews), computer simulation, direct computation, or a combination of these [56]. Qualitative evidence synthesis (QES) refers to all methods involving synthesis of diverse types of qualitative evidence from primary studies [33]. The choice of a QES method will depend on the guideline’s scope and the specific questions asked (see Table 2). For example, thematic synthesis can be used for questions relating to socio-cultural acceptability of an intervention, as it aims to develop descriptive or analytic themes [63]. Meta-ethnography, on the other hand, would be more suitable for questions exploring why and how intervention components work together as it aims to develop new explanations [33, 64].

Synthesis of both qualitative and quantitative evidence (so called “mixed methods”) might be required to answer some guideline questions. For example, quantitative evidence can inform whether the effects of a levy on soft drinks differ for people from different socio-economic backgrounds [35]. Qualitative evidence, on the other hand, can further help to understand the reasons behind these differences. Mixed methods syntheses can involve separate analysis and synthesis of qualitative and quantitative evidence (i.e., segregated design), or a cyclical approach can be taken when the findings from one synthesis inform the next synthesis (i.e., contingent design) [65]. Qualitative and quantitative evidence can be integrated in a guideline in different ways. They may be analysed in a parallel or complementary way (i.e., convergent synthesis) or conducted with one synthesis following and informing the other (i.e., sequential synthesis) [66]. This integration can occur in a single synthesis, or two or more stand-alone reviews may be conducted first and then the findings consolidated in a cross-study synthesis [35].

#### Illustration from a guideline

To inform development of a guideline on protecting, promoting, and supporting breastfeeding practices in healthy mothers with healthy full-term babies [67], a systematic review was conducted [68]. In addition to estimating the overall effects, the review team conducted sub-group analyses to explore the variation of effects based on who was delivering the intervention. The findings showed that the effect on cessation of exclusive breastfeeding at up to 6 months was greater for lay support in comparison with health professionals or mixed support [68]. In addition, QES was conducted on the values and preferences of mothers and the factors influencing acceptability among health workers and stakeholders. It showed that most mothers found that breastfeeding was not adequately taught and reported receiving inconsistent advice from different healthcare workers, which could help explain the observed differences in the effects based on who delivered the intervention. The evidence gleaned from these quantitative and qualitative evidence syntheses was used in structuring and formulating specific guideline recommendations, including those aiming to create an enabling environment through enhanced access to adequate breastfeeding support [67].

### Step 4: assessing the evidence

The next step in guideline development is to assess the quality of each type of contributing evidence, including evidence on effectiveness, as well as evidence addressing broader guideline questions pertinent to complexity. Different approaches can be used based on the question and type of evidence synthesis (see Table 2). For many questions, particularly those relating to effectiveness, the GRADE approach is appropriate [69]. GRADE is designed to rate the certainty of evidence for specific outcomes, which, in a guideline context, reflects the confidence in where the true intervention effect lies relative to a meaningful threshold [70]. Identification of meaningful thresholds across a large number of health (and non-health) outcomes can be challenging for public health and health system guidelines, particularly for global guidelines, whose implementation contexts may vary greatly. Stakeholder consultations can be helpful in identifying meaningful thresholds (see Step 1). The non-null effect can also serve as a relevant threshold in guidelines on public health and health systems interventions, such as the aforementioned example of a levy on soft drinks, as even small effect sizes can be important given the manifold impacts of a levy on the population at large. Guideline panels need to pre-specify the thresholds, as these further inform judgements on specific domains of GRADE, such as inconsistency and imprecision [71].

Extensions to the GRADE approach can be used to assess the quality of other types of evidence considered in a guideline, such as for different criteria of the WHO-INTEGRATE framework (see Table 2) [6]. For example, GRADE-CERQual (Confidence in the Evidence from Reviews of Qualitative Research) has been developed for assessing confidence in findings from QES [39]. Some useful approaches have been developed outside of the frameworks for systematic reviewing and guideline development. For example, Quality Standards for Ethics Analyses (Q-SEA) can be used as a tool to assess the quality of ethics analyses conducted for a guideline [43].

#### Illustration from a guideline

To develop guidelines on the best approaches for strengthening and sustaining Emergency Risk Communication (ERC) capacity, a broad approach to formulating questions, evidence synthesis, and evidence assessment was adopted [72]. The guideline panel chose the SPICE (Setting, Perspective, phenomenon of Interest, Comparison, Evaluation of impact) format for key question development to facilitate identification and synthesis of quantitative, qualitative, and mixed methods evidence, which was expected to be highly relevant to this guideline. The team identified four main methodological streams in evidence synthesis: quantitative methods with comparison groups, quantitative methods using descriptive survey methods, qualitative methods, and mixed methods and case studies. The GRADE approach, a modified GRADE approach, GRADE-CERQual, and a modified GRADE approach combined with GRADE-CERQual were used to assess evidence from these four streams, respectively [72].

### Step 5: developing recommendations

To develop recommendations, guideline panels should consider relevant criteria, such as those published in EtD frameworks [6, 52] along with the evidence collected, synthesised, and assessed for each criterion.

To illustrate how the criteria of the WHO-INTEGRATE framework may be used to develop specific recommendations (see Table 2), let us return to the case of introducing a levy on soft drinks to tackle obesity [6]. Let us assume that during guideline scoping (see Step 1), in addition to focusing on the direct health benefits or harms associated with the intervention, the guideline panel chose the following criteria for in-depth consideration through evidence collection, synthesis, and assessment: the acceptability of the intervention among different groups of stakeholders (e.g., different Ministries, the food industry, the general public), its societal and ecological implications (e.g., changes in social norms in relation to soft drinks and reductions in aluminium and plastic waste), and its impact on health equity, equality, and non-discrimination (e.g., increase in consumption patterns among certain socio-economic groups). The collected and assessed evidence for these criteria will inform specific recommendations. For example, if there is evidence deemed reliable by the guideline panel that shows a positive impact on the environment associated with levy introduction, this may contribute towards making a recommendation in favour of this intervention – given that environmental sustainability is a priority for the guideline panel. Guideline panels will need to use judgement in weighing the importance of the criteria when developing recommendations (e.g., prioritising net societal benefits over intervention acceptability). This prioritisation process should be explicitly documented and reflect the perspectives from all relevant stakeholders (see above) [73].

#### Illustration from a guideline

To develop a guideline on how to safely design and implement sanitation services, the guideline panel conducted a survey of selected global sanitation actors in health, public sectors, sanitation financing, academic institutions, and international and not-for profit organisations to help define the guideline scope and priorities [74]. The team considered evidence for each of the six substantive criteria of the WHO-INTEGRATE framework to formulate guideline recommendations assigning importance to each of these. For each criterion, the evidence was summarised and the rationale was given for making a judgment about how the criterion influenced the recommendation. The meta-criterion, quality of evidence, was applied only in relation to intervention effectiveness, as the panel did not find suitable methods to use it for the other criteria.

## Conclusions

In this paper we describe the process and methods for developing guidelines from a complexity perspective. Public health and health systems interventions often interact with and adapt to the system in which they are implemented; thus in assessing their impact, it is important to consider this wider system. In this step-by-step guide, we particularly emphasise and recommend that guideline panels make investment in the early phase of guideline scoping. This will set the stage for subsequent steps, including timely collection, synthesis, and assessment of evidence. Through explicit consideration of a range of questions in addition to those about intervention effectiveness, taking a complexity perspective will produce guidelines with better informed recommendations and more transparent procedures. Furthermore, by providing an enhanced understanding of the specific circumstances in which interventions work, a complexity perspective can also facilitate local adaptation and implementation of guidelines. This can be achieved through the explicit addition of contextual specifications in the guideline, as well as documentation of the voices and potentially diverging perspectives of key stakeholders. This paper provides general guidance on when and how to take a complexity perspective in guideline development. Although it draws on examples of guidelines on public health and health system interventions and is likely to be of most relevance for guideline panels working on these types of interventions, the described steps can also be applied to guidelines on clinical interventions if a guideline panel thinks that taking a complexity perspective may add value. Indeed, interventions and services in clinical care practice often target complex health issues and are often delivered in complex healthcare systems. For example, it has been shown that use of emergency health services displays characteristics of complex systems, including heavy-tailed distribution and sequences of consultations clustered in time [75]. These call for services that address the whole system rather than focusing on problematic individuals only.

While this paper largely draws on the series of papers from BMJ Global Health, which was developed using a consensus-based methodology and different systematic and other review methods, several areas will benefit from further methodological research and development. Specifically, further work will need to look into the methods of collecting and assessing different types of evidence, such as ethics, financial, and economic analyses (see Table 2). Standardised approaches similar to GRADE and GRADE-CERQual for rating these types of evidence may be helpful. However, such approaches should also be pragmatic to enable rapid application, given that guideline development tends to happen under significant time and resource constraints. There is also a need for further procedural guidance on how to prioritise across a range of EtD criteria in a guideline. Prioritisation can be a challenging process, because of many potentially divergent perspectives [76]. Finally, while there are many published guidelines that have used some of the procedures we describe in this paper, the overall 5-step process has not yet been used and systematically tested in a single guideline. We therefore lack real-world confirmation of the value and feasibility of this approach. However, we are aware of at least one WHO guideline that is currently using this process. As complexity is an evolving topic in public health and health systems research, more examples are needed of guidelines taking such a perspective.

## Supplementary information

**Additional file 1.** WHO-INTEGRATE framework version 1.0: criteria, definitions and example questions (adapted from Rehfuess and colleagues [6]).

## Data Availability

Not applicable.

## Abbreviations

ANC: Antenatal Care
CERQual: Confidence in the Evidence from Reviews of Qualitative Research
ERC: Emergency Risk Communication
EtD: Evidence-to-Decision
GRADE: Grading of Recommendations Assessment, Development, and Evaluation
PerSPEcTiF: Perspective, Setting, Phenomenon of interest, Environment, Comparison, Time, and Findings
PICO: Population, Intervention, Comparison, Outcome
QCA: Qualitative Comparative Analysis
QES: Qualitative Evidence Synthesis
Q-SEA: Quality Standards for Ethics Analyses
SPICE: Setting, Perspective, phenomenon of Interest, Comparison, Evaluation of impact
WHO-INTEGRATE: Word Health Organization INTEGRATe Evidence
